# Calcium renal lithiasis: metabolic diagnosis and medical treatment

**DOI:** 10.1590/S1516-31802013000100008

**Published:** 2013-02-01

**Authors:** Miguel Angel Arrabal-Polo, Miguel Arrabal-Martin, Juan Garrido-Gomez

**Affiliations:** I MD. Resident in training, Urology Department, San Cecilio University Hospital, Granada, Spain.; II MD, PhD. Attending Physician, Urology Department, San Cecilio University Hospital, Granada, Spain.; III MD, PhD. Attending Physician, Traumatology Department, San Cecilio University Hospital, Granada, Spain.

**Keywords:** Kidney calculi, Therapeutics, Metabolism, Diagnosis, Pharmacy, Calcium, Cálculos renais, Terapêutica, Metabolismo, Diagnóstico, Farmácia, Cálcio

## Abstract

Calcium renal lithiasis is a frequent condition that affects the worldwide population and has a high recurrence rate. Different metabolic changes may trigger the onset of calcium stone disorders, such as hypercalciuria, hyperoxaluria, hyperuricosuria, hypocitraturia and others. There are also other very prevalent disorders that are associated with calcium calculi, such as arterial hypertension, obesity and loss of bone mineral density. A correct diagnosis needs to be obtained through examining the serum and urinary parameters of mineral metabolism in order to carry out adequate prevention and treatment of this condition. Once the metabolic diagnosis is known, it is possible to establish dietary and pharmacological treatment that may enable monitoring of the disease and prevent recurrence of stone formation. Some advances in treating this pathological condition have been made, and these include use of sodium alendronate in patients with calcium renal lithiasis and osteopenia/osteoporosis, or use of a combination of a thiazide with a bisphosphonate. In summary, calcium renal lithiasis often requires multidrug treatment with strict control and follow-up of patients.

## RESUMO

Litíase renal cálcica é uma doença comum que afeta a população no mundo todo e tem alta taxa de recorrência. Diferentes alterações metabólicas podem desencadear o aparecimento de distúrbios de pedras de cálcio, como hipercalciúria, hiperoxalúria, hiperuricosúria, hipocitratúria e outros. Existem também doenças altamente prevalentes associadas à doença de cálculo de cálcio, como hipertensão, obesidade e perda de densidade óssea mineral. Para realizar prevenção e tratamento adequados, é necessário diagnóstico correto, examinando o metabolismo mineral sérico e urinário. Depois de conhecer o diagnóstico metabólico, é possível estabelecer um tratamento dietético e farmacológico que permita controlar a doença e prevenir a recorrência de cálculos biliares. Há alguns avanços no tratamento dessa doença e incluem o uso de alendronato de sódio em pacientes com nefrolitíase de cálcio e osteopenia/osteoporose, ou a combinação de um tiazídico com um bifosfonato. Em resumo, litíase renal cálcica exige, muitas vezes, um tratamento multidroga com rigorosos controle e acompanhamento de pacientes.

## INTRODUCTION

Calcium kidney stones are an important urological and nephrological pathological condition that affects a high percentage of the population during life. The information available on their etiology, clinical presentation, diagnosis and treatment (medical and non-medical) is very extensive and therefore, in this review article, we will address their metabolic diagnosis and medical treatment, particularly by reviewing published papers in the most important databases.

PubMed, Lilacs, Embase and Cochrane Library were searched for systematic reviews and practice guidelines, using the MeSH terms Kidney calculi AND Calcium AND Metabolism with the following limits: review, clinical trial, randomized clinical trial, diagnostic tests and evaluation studies. We found a total of 533 articles in PubMed, 43 articles in Embase, 26 articles in Cochrane and 6 in Lilacs. Finally, we selected a total of 63 articles from PubMed, 6 from Cochrane and 3 from Embase. The 6 articles and 3 articles from Cochrane and Embase were also among the 63 articles used from PubMed (Table 1).

**Table 1.** Search performed using the terms “Calcium stones AND Metabolism” in the principal databases, and numbers of articles selected

Databases	Terms used	Limits	Results	Relevant results
PubMed	Kidney calculi Calcium Metabolism	Clinical trial Review Randomized clinical trial Evaluation studies Diagnostic tests	533	63
Cochrane Library	Kidney calculi Calcium Metabolism	Clinical trial Review Randomized clinical trial Evaluation studies Diagnostic tests	26	6
Embase	Kidney calculi Calcium Metabolism	Clinical trial Review Randomized clinical trial Evaluation studies Diagnostic tests	43	3
Lilacs	Litiasis renal Calcio Metabolismo	Ensayo clínico Revisiones Ensayo clínico aleatorizado Estudios evaluación Testes diagnósticos	6	0

## EPIDEMIOLOGY

Renal lithiasis is a very frequent pathological condition among the worldwide population, with varying incidence according to distinct geographical regions. Calcium-based stones, both in oxalate and in phosphate form, prevail in developed countries, while infectious lithiasis remains the main cause of this condition in developing countries.^1^ Renal lithiasis occurs at least once in life in 15% of Caucasian men and in 6% of women, with recurrence of around 50%.^2^

In most cases (around 75%), stones have a calcium composition, and they generally present in the form of calcium oxalate, whereas other types of lithiasis are less frequent.^2^ Increased frequency of calcium phosphate stones has been observed over the last few decades, and above all among women, with metabolic and bone-derived diseases.^3^

The geographical variability in the prevalence of lithiasis is also noteworthy, since in some zones it is very high, while in others it is nonexistent.^4^ In general, urinary lithiasis has a mean incidence of 0.5-1%, and an annual prevalence of 4-5%.^5^

## CLINICAL AND METABOLIC DIAGNOSIS

### Clinical diagnosis

Renal colic is the most common clinical presentation of calcium renal lithiasis and leads to a high admission rate in the emergency rooms of hospitals. It is very important to objectively describe the type of pain, quantify it, determine the origin of irradiation and collect any personal and family histories relating to calcium renal lithiasis. Patients should be asked about their dietary patterns, fluid intake, use of medication and type of physical activity that they perform.^6^ Simple abdominal radiography and ultrasonography are the imaging techniques usually ordered at the beginning of the study, although abdominal computed tomography (CT) is the gold standard imaging technique (Table 2) for diagnosing and classifying renal lithiasis. Today, and thanks to new imaging techniques such as dual-energy computed tomography, it is possible to obtain a sufficiently accurate diagnosis to be able to differentiate calcium stones from uric acid, or from other types with less frequent chemical compositions.^7^

**Table 2.** Summary of highlights of each of the sections covered in the review along with their level of scientific evidence; main results and strategies evaluated in the review.

	Level of evidence
Clinical and metabolic diagnosis	
CT scan is the “gold standard” for diagnosing calcium stones	1a
Randall’s plaque is important in development and pathogenesis of calcium oxalate stones	
Stone composition is well described with infrared spectroscopy and X-ray diffraction	2
24-hour urine test is recommendable for diagnosing metabolic alterations in calcium stones	2a
Hypercalciuria is the most frequent alteration in metabolic studies	2a
Calcium lithiasis and its relationship with other prevalent diseases	
Arterial hypertension and obesity are related to calcium stones	3
Bone mineral density loss and calcium stones are two pathological conditions correlated with genetics and calcium and phosphorus metabolism	3
Medical management and prophylaxis of calcium lithiasis	
Fluid intake of more than 2 liters/day is recommended	1b
Calcium intake should be normal (approximately 1000 mg/day)	1b
Restriction of oxalate, animal protein, salt and purine intake is recommended	1b
Prevention and pharmacological treatment	
Potassium citrate prevents calcium stones in patients with hypocitraturia and acid pH	1b
Allopurinol is indicated for calcium stones with hyperuricosuria and increased serum uric acid	2a
Thiazides have been demonstrated to decrease calciuria and prevent new episodes of calcium stones	1a
Bisphosphonates form a new therapy for patients with calcium stones and bone mineral density loss that improves both of these conditions	3

### Metabolic diagnosis

Lithiasis composed of calcium oxalate is the most frequent type, followed by calcium phosphate. Numerous studies have highlighted the significance that Randall’s plaque has in relation to development and pathogenesis of calcium oxalate lithiasis (Table 2), thanks to the use of intraoperative biopsies.^8^^,^^9^ It has even been possible to demonstrate that the singularity of the renal papilla depends on the type of renal calcium stone former, i.e. whether it is oxalate or phosphate.^10^ Microscopic lesions on the renal papilla are the main cause of formation of monohydrate calcium oxalate stones, while the location of such lesions and their characteristics will in many instances determine the final morphology of the renal calculus.^11^ Even the presence of lesions in kidney tubular cells may favor aggregation and growth of crystals, which eventually leads to formation of calcium stones.^12^

Calcium stone formation is a complex process that involves numerous metabolic, anatomical and physiopathological mechanisms. Occurrences of supersaturated states of crystallization with the capacity to precipitate in urine are a key factor in calculus formation. The saturation state of a substance is expressed as the ratio between a given substance and its solubility variable. The supersaturated state of the calcium oxalate is unrelated to the urinary pH; however, calcium phosphate saturation increases significantly when the urinary pH changes from 6 to 7.^13^^,^^14^

Metabolic disorders are, beyond any doubt, key factors in the formation and persistence of calcium lithiasis. It is therefore advisable to conduct a complete metabolic study on patients with histories of calcium lithiasis, multiple calcium lithiasis or hard-to-treat calcium lithiasis; and on calcium lithiasis-forming children, single kidney patients and occurrences of intestinal disorders, bone disorders or nephrocalcinosis.^15^ Metabolic studies include analysis on the calculi, since each occurrence of lithiasis comprises in itself a certain clinical form of lithiasis disease. Firstly, the macroscopic structure of the calculi, before and after fragmentation, should be examined in order to identify their exact composition through infrared spectroscopy and X-ray diffraction along with other electronic scanning microscopy techniques. On the other hand, chemical analysis of calculi is an inexact method that has now become obsolete.^16^

Regarding blood and urine results, tests should be ordered when it is necessary to know the blood levels of calcium, phosphorus, sodium, potassium, chlorine, magnesium, urea, creatinine and uric acid. In 24-hour urine tests, it is recommendable to determine the urea, creatinine, uric acid, oxalate, citrate and magnesium levels, and to establish the diuresis volume, pH and urinary density. A fasting urine test to assess the levels of calcium, creatinine, oxalate and citrate is also recommendable.^5^^,^^7^^,^^8^^,^^9^^,^^10^^,^^11^^,^^12^^,^^13^^,^^14^^,^^15^^,^^16^^,^^17^^,^^18^^,^^19^ The analysis should be extended in order to determine the levels of bone resorption markers in cases of nephrocalcinosis, suspected hyperparathyroidism, fasting hypercalciuria or previous treatment for loss of bone mineral density. Blood tests should detect markers for intact parathyroid hormone (iPTH), vitamin D, alkaline phosphatase, osteocalcin and beta-crosslaps. Furthermore, any metabolic study conducted on patients with recurrent calcium renal lithiasis and altered bone remodeling markers can be complemented by performing bone densitometry to objectively assess bone mineral density.^20^

Metabolic studies reveal a series of alterations that can affect the calcium stone composition, and which can be summarized as follows:

#### Hypercalciuria

Hypercalciuria is, for many reasons, the most important metabolic risk factor for formation of calcium lithiasis. Hypercalciuria is the most frequently detected metabolic abnormality, in 35-65% of patients with stones; the relation between urinary calcium levels and occurrences of Randall’s plaques is another factor;^19^^,^^21^ also, a genetic and molecular relationship with the presence of calcium in urine could be regarded as a further factor.^22^^,^^23^ Hypercalciuria is defined, according to different studies, as excretion in urine of more than 260 mg of calcium in 24 hours (or 4 mg of calcium per kg/day). In clinical practice, it is also valuable to regard hypercalciuria as excretion of more than 250 mg/day in women and 300 mg/day in men.^5^^,^^9^^,^^24^ Although the term idiopathic hypercalciuria has been used for years to refer to increased urinary calcium levels, it is more accurate to talk about distinct types of hypercalciuria, on the basis that the calcium transport defect may be localized in the gastrointestinal tract, bones or kidneys. Thus, hypercalciuria can be classified into three groups: absorptive hypercalciuria; excretory, or renal, hypercalciuria; and reabsorptive hypercalciuria.^5^^,^^19^^,^^20^^,^^21^^,^^22^^,^^23^^,^^24^^,^^25^

- Absorptive hypercalciuria: Three types of hypercalciuria have been defined and are characterized by having a calcium/creatinine ratio in urine of less than 0.11 during fasting, and less than 0.22 when there is an overload of calcium in the diet. They differ from each other in that type 1 has high calcium excretion in urine even with a restricted calcium diet; in type 2, lower calcium levels in urine are detected following restriction of calcium in the diet; and a deficit of serum phosphate is detected in type 3.^26^^,^^27^
- Excretory hypercalciuria: This occurs as a result of failure of renal tubular reabsorption of calcium. These patients have calcium/creatinine ratios greater than 0.11 while fasting, and higher than 0.22 after calcium intake. Furthermore, iPTH is found to be within the normal range.^19^
- Reabsorptive hypercalciuria: This results from increased bone resorption, basically in relation to primary hyperparathyroidism. Calcium/creatinine ratios are found to be high while fasting and after calcium overloads greater than 0.11 and 0.22, respectively, as well as the iPTH levels.

#### Hyperoxaluria

Hyperoxaluria is defined as urinary excretion of oxalate higher than 40 mg/day. Increased oxalate excretion in urine seems to be associated with formation of monohydrate calcium oxalate stones, which is a metabolic risk factor in this type of lithiasis disorder. As with hypercalciuria, various types of hyperoxaluria can be distinguished. Primary hyperoxaluria (type 1 and type 2) can be found in patients with enzyme deficits resulting from mutations of a variety of genes, and it involves anomalous and incomplete degradation of oxalates that accumulate in urine before their excretion. However, this condition is usually infrequent.^28^

Enteric hyperoxaluria is the most frequent disorder, and results from malabsorption of fats. Since these fats cannot be absorbed, they bind to calcium and diminish the formation of intestinal calcium-oxalate complexes, and thus increase the intestinal absorption of oxalates. Dietetic hyperoxaluria is caused by an increase in the intake of oxalate-rich food, or excessive intake of vitamin C.^29^

Moreover, the bacterium *Oxalobacter formigenes* has recently been described as the causative agent of oxalate absorption at intestinal level. It seems that low levels of this microorganism lead to increased oxalate levels, since oxalate metabolism diminishes because of this bacterium.^30^

In the past, bariatric surgery played a significant role in the formation of oxalate calculi, since jejunum-ileum shunts would lead to high levels of hyperoxaluria through increased abdominal absorption of oxalates. This has now been replaced by other techniques with fewer side effects on metabolism.^31^

#### Hypocitraturia

Urinary citrate is a recognized inhibitor of oxalate and calcium phosphate stones, through avoiding the formation of the nucleus of stone, its growth and its aggregation.^32^^,^^33^^,^^34^ Hypocitraturia can be defined as urinary excretion of citrate less than 320 mg/day. Citrate has a triple protective effect, since it can bind to calcium to prevent formation of complexes, alkalinize urine and have a direct inhibitory effect. Urinary citrate is reabsorbed under states of acidosis, with the result that its urinary excretion decreases. In addition, hypocitraturia has been correlated with chronic diarrhea, extenuating physical exercise and excessive intake of animal proteins.^19^^,^^35^

#### Hyperuricosuria

Hyperuricosuria is defined as urinary excretion of more than 600 mg/day of uric acid. Excessive presence of this ion in the urine increases heterogeneous nucleation with oxalate calculi, and therefore it plays an influential role in formation of oxalate-calcium calculi. In general, increased urinary excretion of uric acid results from high intake of purines, although it can also be associated with other pathological conditions, such as gout, myeloproliferative diseases, myeloma etc.^5^^,^^36^^,^^37^

#### Hypomagnesuria

The disorder of hypomagnesuria is an infrequent cause of formation of calcium oxalate stones. It can be detected in around 1% of patients and is associated with intestinal inflammatory disease.^5^^,^^36^

## CALCIUM LITHIASIS AND ITS RELATIONSHIP WITH OTHER PREVALENT DISEASES

### Arterial hypertension and calcium lithiasis

Numerous studies have analyzed the relationship between lithiasis and arterial hypertension.^38^^,^^39^ It seems that increases in circulatory volume and arterial pressure lead to decreased sodium reabsorption at the level of the proximal tubules, which results in diminished calcium reabsorption and subsequently, increased calciuria (Table 2). High sodium intake in these patients’ diet is another factor to bear in mind, since this increases the urinary excretion of calcium.^40^^,^^41^

### Obesity and calcium lithiasis

A variety of studies in the literature have shown that there is a relationship between body mass index and kidney stones. It has been increasingly recognized that obesity is associated with uric acid-derived lithiasis, although the prevalence of calcium lithiasis can also be high in these patients as a result of the process of hyperuricosuria, which is, as noted earlier, a metabolic risk factor for calcium lithiasis.^42^ It has been observed that obese patients who underwent bariatric surgery have higher levels of oxaluria, which may result in higher incidence of calcium lithiasis.^43^

### Loss of bone mineral density and calcium lithiasis

The involvement of the kidneys in regulating phosphorus-calcium metabolism through excretion and reabsorption of calcium and phosphorus, and in mediating 1.25 OH vitamin D and iPTH, is well known. It has been shown that the incidence of calcium renal lithiasis is higher in patients with losses of bone mineral density, while calciuria levels are also found to be high.^44^ Some patients present genetic predisposition towards both calcium renal lithiasis and loss of bone mineral density.^45^ Higher incidence of pathological fractures is also seen in osteoporosis patients with calcium renal lithiasis.^46^^,^^47^ With regard to clinical data, our group recently found that patients with recurrent calcium renal lithiasis have higher levels of bone remodeling markers, caused by greater loss of bone mass (revealed though bone densitometry) and by higher levels of calciuria in 24-hour tests.^20^ It is worth bearing in mind the relationship between calcium renal lithiasis and osteopenia/osteoporosis (Table 2), especially in patients with recurrent hypercalciuria and elevated bone marker levels, given that these patients could benefit from treatments aimed at improving bone mineral density and reducing the recurrence of stone formation.^47^^,^^48^^,^^49^^,^^50^

### Diabetes mellitus and calcium lithiasis

So far, there is no clear evidence of higher prevalence of calcium lithiasis in patients with diabetes mellitus. Only higher incidence of uric acid lithiasis has been detected in these patients, along with increased urinary excretion of uric acid.^51^^,^^52^

## MEDICAL MANAGEMENT AND PROPHYLAXIS OF CALCIUM LITHIASIS

### Diet

#### Fluid intake

In general, fluid intake of more than two liters per day is recommended for all patients, since this decreases saturation states by making urine more diluted and lowering the concentrations of crystallizable substances. Low-mineral water, especially with low sodium and calcium levels, is recommended for consumption.^53^

#### Calcium intake in the diet

Some years ago, a calcium-restricted diet was recommended, especially among patients presenting both calcium lithiasis and hypercalciuria. However, it has been noticed that restricted-calcium diets result in higher absorption of enteric oxalate and increased loss of bone mineral density, thereby leading to osteopenia/osteoporosis. A calcium-restricted diet is only recommendable for patients without any risk of loss of bone mineral density or of type 2 absorptive hypercalciuria. For other patients, a normal intake of 1000 mg of calcium per day is recommended in order to prevent both bone demineralization and increased intestinal absorption of oxalate.^54^^,^^55^

#### Fiber-rich food

Fiber intake leads to a change in bowel transit that diminishes the absorption of both calcium and oxalate and thus reduces the incidence of calcium lithiasis. Therefore, moderate fiber intake is recommended for patients with recurrent lithiasis. However, there is not enough scientific evidence to corroborate the benefits of this measure.^53^^,^^55^

#### Oxalate restriction

Restriction of the intake of oxalate-rich food is recommended for patients with hyperoxaluria levels greater than 0.45 mmol/24 h, as a way of diminishing oxalate absorption in the bowel. This restriction includes rhubarb, chocolate, spinach, walnuts, black tea, etc.^53^ Moderate consumption of vitamin C is also recommendable, since this leads to natural conversion of ascorbate to oxalate, which increases the levels of this substance, and hence those of oxaluria.^55^

#### Restriction of protein intake

It is advisable to avoid high protein intake, since this raises calciuria and oxaluria levels. This is therefore harmful for patients with recurrent calcium lithiasis. High protein intake also diminishes urinary citrate levels and lowers urinary pH.^53^^,^^54^^,^^55^^,^^56^^,^^57^

#### Restriction of salt and purine-rich food

In general, it is advisable to restrict the intake of salt and purine-rich foods among patients with hypercalciuria, or in cases of supersaturation of urinary calcium, since this it increases natriuresis and consequently, calciuria.^55^ Intake of purine-rich foodstuffs (oily fish, meat offal etc.) should also be restricted, since they increase uricosuria, which may be damaging for patients with calcium oxalate lithiasis and hyperuricosuria, as it favors heterogeneous crystallization.^55^^,^^57^

## PREVENTION AND PHARMACOLOGICAL TREATMENT

### Potassium citrate

Administration of potassium citrate increases both urinary pH and citrate levels in urine. Citrate is a crystallization inhibitor in urine, and it reduces supersaturation of both oxalate and calcium phosphate by inhibiting the aggregation and growth of such crystals (Table 2). It is especially recommended for patients with calcium lithiasis accompanied by hypocitraturia or hyperuricosuria in acid pH. Urinary pH should be monitored to avoid excessive alkalinization.^53^^,^^57^^,^^58^

### Allopurinol

Administration of allopurinol is recommended in cases of calcium oxalate lithiasis (Table 2), since it reduces production of endogenous uric acid and hence the presence of uric acid in urine.^53^

### Phosphate

Phosphate administration is indicated for patients with calcium lithiasis and type 3 absorptive hypercalciuria. In such patients, a phosphate deficit may lead to increased calcium levels in urine.^27^

### Pyridoxine

Although there is no clear evidence of beneficial effects from pyridoxine supplementation, this is indicated for patients with calcium oxalate lithiasis and hyperoxaluria.^5^

### Thiazides

Thiazides (hydrochlorothiazide and indapamide, among others) produce an increase in tubular reabsorption of calcium, which diminishes calciuria.^53^ This drug is indicated for the majority of patients with recurrent calcium lithiasis and hypercalciuria (Table 2), since it has been reported to diminish the recurrence of lithiasis and improve the monitoring of this condition.^59^ It has also been noticed that use of thiazides in cases of residual lithiasis resulting from instrumental management of the disorder stabilizes it and prevents further calculus formation.^60^ Thiazides can currently be regarded as an efficient treatment for preventing recurrent lithiasis, and for treating residual lithiasis processes that cannot receive instrumental management.^53^^,^^57^^,^^59^^,^^60^

### Bisphosphonate

Treatment of calcium renal lithiasis with bisphosphonate is novel but, despite the sparseness of studies on its indication, fasting and 24-hour tests have reported decreases in calciuria among patients with calcium renal lithiasis and loss of bone mineral density, following this treatment.^61^^,^^62^ Furthermore, some studies have reported that treatment based on sodium alendronate, alone^50^ or in combination with indapamide,^63^ provides improvements for conditions of recurrent lithiasis and loss of bone mineral density (Table 2).
